# Short and Long-Term Effects on Academic Performance of a School-Based Training in Self-Regulation Learning: A Three-Level Experimental Study

**DOI:** 10.3389/fpsyg.2022.889201

**Published:** 2022-05-12

**Authors:** Ellián Tuero, José Carlos Núñez, Guillermo Vallejo, María Paula Fernández, Francisco Javier Añón, Tânia Moreira, Joana Martins, Pedro Rosário

**Affiliations:** ^1^Department of Psychology, University of Oviedo, Oviedo, Spain; ^2^Department of Applied Psychology, University of Minho, Braga, Portugal

**Keywords:** self-regulated learning strategies, intervention, academic performance, follow-up effects, multilevel analysis

## Abstract

An experimental study was designed to analyze the effect of school-based training in self-regulation learning strategies on academic performance (Mathematics, Sciences, Language, and English). Class-level variables (i.e., gender, the teacher’s teaching experience, class size) were considered and the effects of the intervention were measured at the end of the intervention and 3 months later. A sample of 761 students from 3rd and 4th grades (356 in the control condition and 405 in the experimental condition), from 14 schools, participated in the study. Data were analyzed using three-level analysis with within-student measurements at level 1, between-students within-classes at level 2, and between-classes at level 3. Data showed a positive effect of the intervention on student performance, both at post-test (*d* = 0.25) and at follow-up (*d* = 0.33) considering the four school subjects together. However, the effect was significant just at follow-up when subjects were considered separately. Student performance was significantly related to the students’ variables (i.e., gender, level of reading comprehension) and the context (teacher gender and class size). Finally, students’ gender and level of reading comprehension, as well as the teacher’s gender, were found to moderate the effect of the intervention on students’ academic performance. Two conclusions were highlighted: first, data emphasize the importance of considering time while conducting intervention studies. Second, more teaching experience does not necessarily translate into improvements in the quality of students’ instruction.

## Introduction

Winne and Hadwin (2013) have identified three challenges associated with task execution. Students may face difficulties in (i) fully understanding the characteristics of the learning task at hand (this aspect is particularly relevant, because understanding the task is expected to guide the planning for its development and inform the selection of study strategies, monitoring, and adaptation of the learning process); (ii) using the learning techniques or strategies recently acquired to accomplish the task; and (iii) transferring the learning strategies acquired in one context to other contexts. These are some of the reasons why there is a general agreement that self-regulated learning (SRL) plays an essential role not only in improving academic performance but also in the overall development of students throughout their lives, regardless of the context (Lüftenegger et al., 2012; Artuch-Grade et al., 2017; Venitz and Perels, 2018; Jansen et al., 2019; Martínez and Valiente, 2019; Chu et al., 2020; Theobald, 2021).

Previous research has generally indicated a positive relationship between the use of SRL strategies and improvements in attentional processes, planning and self-evaluation skills (Boekaerts and Corno, 2005; Cerezo et al., 2019), consistency and task persistence (Nota et al., 2004), perceived competence for schoolwork (Núñez et al., 2013), improvements in problem-solving processes (Verschaffael et al., 2010; Bol et al., 2015), school performance (Cleary and Platten, 2013), and academic success in general (Núñez et al., 2011). Narrowing the focus of the relationship between SRL and academic performance down to particular content domains, research has found positive effects of this relationship in mathematics (Fuchs et al., 2003; García et al., 2019), science (Guthrie et al., 2004), writing (Fidalgo et al., 2008; Rosário et al., 2017a,b, 2019), and reading (Spörer and Schünemann, 2014).

Self-regulated learning may be understood as an active, strategic, cyclical, and recurring process (Zimmerman, 2008) in which students are expected to set learning goals and manage behaviors, thoughts, and emotions, adapting the latter if necessary to attain the former (Pintrich, 2000; Zimmerman, 2011; Rosário et al., 2012). Thus, SRL implies the systematic development of cognitive, metacognitive, motivational, and behavioral processes, as well as the ability to adapt strategies to various contexts and attain the goals set (Pintrich, 2000; Efklides, 2011; Hederich-Martínez et al., 2016; Cerezo et al., 2017; Cho et al., 2017; Frazier et al., 2021). In an academic context, metacognition is a process closely related to SRL, referring to the ability to reflect, understand, and control one’s own thoughts. Metacognition was defined by Flavell (1979) as knowledge concerning one’s own cognitive processes and products, and is generally understood as involving knowledge, the monitoring, and the control of those processes (McCormick, 2003). However, self-regulation requires more than metacognitive knowledge and skill. Self-regulation involves an underlying sense of self-efficacy and personal agency and the motivational and behavioral processes to put these self-beliefs into effect. In fact, despite showing robust metacognitive knowledge, students may fail to activate and sustain their efforts and regulate sources of personal influence (e.g., managing emotions and environmental distractors) toward a self-set goal (Zimmerman and Moylan, 2009). The work of Winne and Perry (2000), which stresses that the optimized use of metacognitive strategies is critical to the effective regulation of study, is consistent with the latter proposition. For example, students who regularly check the strategies used to overcome distractors while studying (e.g., putting the phone in airplane mode to limit text messages and phone call interruptions during study time) are likely to attain their goals and improve performance.

Data from recent meta-analyses (e.g., Dignath et al., 2008; Donker et al., 2014; De Boer et al., 2018) show that the effect of self-regulatory skills on academic performance is statistically significant, with a moderate effect size, between 0.50 and 0.60. For example, a meta-analysis by Dignath et al. (2008) examined the effectiveness of self-regulatory training among elementary-school students. One of the topics used to select the studies for the meta-analysis was the type of strategies trained (i.e., cognitive, metacognitive, or motivational). For example, studies using metacognitive strategies addressed second-order cognitions aimed at controlling, monitoring, and evaluating learning and cognitive tasks. A detailed analysis of the findings regarding the use of metacognitive reflection strategies showed that interventions with the highest effect sizes equipped students with knowledge about strategies and provided them with opportunities to apply the learning strategies in class activities.

However, and despite the promising results found (Rosário et al., 2020), to the best of our knowledge, there are still some important unresolved questions. For example, there are conflicting data about the effects of interventions beyond the post-test (e.g., De Boer et al., 2018). The meta-analysis by De Boer et al. (2018) found that the positive effect of interventions was maintained and even increased in the post-test, but these authors warned that the results may have been related to singularities, such as particular learning domains, student variables, or the types of cognitive and metacognitive strategies trained. Another aspect that has shown mixed results is related to the age of the participants. Dignath et al. (2008) found that the effect of intervention programs was more effective for students in the early school grades, but the meta-analysis by Donker et al. (2014) reported no statistically significant differences related to student age. Moreover, a few studies on the relationship between training in SRL strategies and academic performance have examined the impact of this training on several subjects simultaneously, while assessing the same students. Also, the data are scarce on the way by which class variables can affect the relationship between SRL and academic performance either after an intervention (post-test) or in the longer term (follow-up).

So, drawing on the recommendations from previous meta-analyses, an experimental study was conducted (with control and experimental groups) in an authentic learning context on Spanish language (e.g., reading, writing, comprehension and composition of texts). This intervention (the Rainbow Program) was grounded in the theoretical framework of the social-cognitive learning model from Zimmerman (2011) and followed a metacognitive approach to train SRL strategies at the elementary-school level (8 and 9 years old). See information on this framework in the section “Procedure and Intervention Program.”

A 12-session intervention program was delivered on a weekly basis to a large sample of elementary students from the third and fourth grades. Reasons for focusing the intervention on these two intermediate elementary-school grades were twofold. First, educational interventions addressing children enrolled in these grades are limited; the data from the current research are expected to provide information on the educational needs of students at an early stage and therefore to help establish school-based interventions on SRL. Second, the effect size of the intervention may be related to the age of the students, and therefore the resulting data are expected to add to the literature. The current study collected data at three measurement time points: pre-test, post-test, and follow-up (3 months after the intervention). The third set of data was gathered because one of our objectives was to analyze the maintenance of the reported behavioral changes 3 months post-intervention. The large sample used allowed us to analyze the effect of the intervention on performance at a student level but also to examine the effect of class-level variables (i.e., teacher’s gender, teacher’s teaching experience, and class size). To our knowledge, although some studies have analyzed the effect of class-level variables (e.g., Stoeger et al., 2014; Lüftenegger et al., 2016), there is limited information about how these class-level variables conditioned the effects of an SRL intervention program on students’ performance. The present study is also expected to contribute to deepening our understanding of the role played by class-level variables on the effectiveness of such interventions. To that end, data will be analyzed using a multilevel strategy (i.e., a three-level analysis with within-student measurements at level 1, between students within classes at level 2, and between classes at level 3).

In summary, a controlled study was run to examine the effect of training in SRL strategies on academic performance among students from the third and fourth grades. Data were collected prior to and at the end of the intervention, as well as 3 months post-intervention. Moreover, the effects of the intervention on performance were evaluated while considering the effects of class-level variables such as teacher’s gender, teacher’s teaching experience, and class size. The elementary-school classes were randomly assigned to the experimental condition (participants were delivered an SRL intervention) or to the control group (participants followed the usual school curriculum without any interventions). So, the effect of the intervention was measured regarding three aspects: (a) once the intervention had finished, (b) over the long term, and (c) in light of the effect of class-level variables. Data were analyzed using a multilevel strategy (three-level analysis with within-student measurements at level 1, between students within classes at level 2, and between classes at level 3).

The study addresses the following questions:

(1) Does the incorporation of the Rainbow Program into regular instruction significantly improve fourth- and fifth-grade students’ academic achievement? Based on the results of the previous investigation (e.g., Dignath et al., 2008; Donker et al., 2014), it is hypothesized that, once the intervention is finished, students in the experimental group (Spanish curriculum + SRL training) will show higher performance in the four curricular areas than those in the control group (just Spanish curriculum). However, as suggested by the data from Jansen et al. (2019), only part of the effect of SRL intervention on achievement is expected to be mediated by the SRL activity; therefore, the effect size of the SRL intervention on performance is expected to be small or modest.

(2) Do the positive effects of the training last for 3 months after the intervention? In the meta-analysis by De Boer et al. (2018), most of the studies that included third- or fourth-year students showed that the gains in the post-test were maintained or increased in the follow-up. So, it is hypothesized that the effect size for the follow-up (3 months post-intervention) is similar or even slightly larger than the effect size for the post-test. The gains are expected to be maintained, or even increased, because the students have had the opportunity to practice the skills acquired through their involvement in the SRL activities developed in class for 3 months.

(3) To what extent the effects of the intervention are moderated by student variables and context variables? Prior data has shown that teacher characteristics (e.g., Wayne and Youngs, 2003) and class size (e.g., Krueger and Whitmore, 2001) show positive relationships with student performance, although data from recent studies indicate low consistency (e.g., Blömeke and Olsen, 2019). However, regardless of the mixed results found, none of the above studies examined the effects of these variables at the class level. With regard to student variables, the available data are also inconclusive. Whereas some studies indicate that high achievers benefited more than low achievers from intervention programs, others suggested a Matthew effect as a plausible explanation for the data (e.g., Sontag and Stoeger, 2015; Otto and Kistner, 2017). Therefore, the differences found for the three context variables (i.e., gender of the teacher, teaching experience, and class size) and for the three variables of the student (i.e., gender of the student, level of reading comprehension, and SRL strategies), alongside the limited data on the role played by these variables in the outputs of the intervention, do not permit the establishment of conditional hypotheses. Therefore, from an exploratory perspective, we aimed to examine the potential mediating roles of the personal variables (i.e., gender of the student, level of reading comprehension, SRL strategies) and the contextual variables (i.e., gender of the teacher, teaching experience, class size) on the effect of the intervention on students’ academic performance.

## Materials and Methods

### Participants

An initial sample of 915 students from the third (*n* = 486; 53.1%) and fourth (*n* = 429; 46.8%) grades participated in the study. These students were enrolled in 50 classes of 14 public (*n* = 607; 66.3%) and charter schools (*n* = 308; 33.6%) in the Principality of Asturias in the North of Spain. This sampling was non-probabilistic and incidental. The 50 classes enrolled were randomly assigned either to the experimental or control group. The mean number of students per class was 22.30 (*SD* = 4.24). Most of the teachers were female (75.2%), and with extensive teaching experience (*M* = 22.30; *SD* = 12.22). For various reasons (e.g., change of residence over the period of the intervention, absence from class on the day of the assessment, special educational needs), of the 915 students, 154 did not participate in the study. Finally, 761 students aged between 8 and 11 years (*M* = 8.81; *SD* = 0.73) were included in the analyses (356 in the control condition and 405 in the experimental condition). No statistically significant differences were found for gender (49.6% girls; experimental group 49.9% girls; control group 49.4% girls). The majority of the families of these children were from medium-to-high socioeconomic backgrounds, living in urban areas.

### Measures

#### Strategies for Self-Regulated Learning

Self-regulated learning strategies were assessed with the Inventory of Processes of Self-Regulation of Learning (IPAA; Rosário et al., 2007). The IPAA is based on the socio-cognitive model of Zimmerman (2000; 2008; 2011). It consists of nine items measuring the three phases of the process of SRL: planning (e.g., I make a plan before starting to work. I think about what I’m going to do and what I will need in order to do it), execution (e.g., During class and when I study at home, I think about specific parts of my behavior to change to achieve my objectives), and evaluation (e.g., I keep and study my corrected work to see where I went wrong and to understand what I have to do to improve). Item responses used a Likert-type format with five alternatives (1 = never, 5 = always). The IPAA has been adapted and used at different ages and school levels (elementary, high school, and college), showing adequate psychometric properties (e.g., Rosário et al., 2012 and Núñez et al., 2013, in elementary; Rosário et al., 2012, in high school; and Rosário et al., 2015 and Cerezo et al., 2019, in college). In the present study, the Cronbach’s alpha is 0.80, indicating satisfactory reliability.

#### Reading Comprehension

The students’ ability to understand texts was assessed using the Evaluation Battery for Reading Processes-Revised (PROLEC-R; Cuetos et al., 2007). This is a widely used test with robust levels of reliability and validity (e.g., Goswami et al., 2011). For the purposes of this study, we used the text comprehension subtest. This subtest is made up of four short texts of increasing difficulty and questions about them that are both direct and inferential.

#### Academic Performance

In the Spanish educational system, student performance is evaluated three times each school year. The assessment tests used in class are non-standardized knowledge evaluation tests, although they are similar for students of the same grade level. These content-domain tests include tasks of a distinct nature and complexity (e.g., problem-solving, investigative, or practical tasks). The regular teachers were asked to provide their students’ scores for each of the four subjects (science, Spanish language, English language, and mathematics) at each of the three evaluation time points (pre-test, post-test, and follow-up). The rating used a 5-point scale (1 = minimum, 5 = maximum).

### Design and Procedure

#### Design

A classroom-based randomized trial (CRT) was used to minimize contamination from the application of the treatment program in situations in which experimental students routinely interact (at the class level). It should be noted that the intervention program was embedded in school practices; consequently, it was infeasible to randomize individual students. Moreover, CRT is a natural design choice to respond to current research questions. Specifically, in this study, pre-existing groups of students (classes in our study), rather than individual students, were randomly assigned to either the intervention or control condition (standard treatment), and students regardless of condition were assessed for four dependent variables (i.e., science, Spanish language, English language, and mathematics) on three successive occasions [baseline, post-intervention, and follow-up (3 months after intervention)]. We examined baseline differences in text comprehension and SRL because students were not randomly assigned to classes and the effectiveness of the intervention may have been conditioned by their initial skills in text comprehension and SRL. The results showed no statistically significant differences in SRL strategies (*p* = 0.373) but statistically significant differences in text comprehension, although with a small effect size (*d* = 0.25). Therefore, both variables were included as covariates.

#### Training for Implementers

Two weeks prior to the beginning of the study, a training course of four 3-h sessions was delivered separately to the 41 participating teachers, regardless of treatment conditions. The course had two modules of 6 h each. The first presented and discussed the general SRL framework (e.g., social-cognitive theoretical framework, promotion of SRL learning in the classroom), while the second addressed the organization of the course (e.g., schedules, training on questioning to trigger student reflection and metacognitive reasoning), the assessment process, and the protocol for each session.

#### Treatment Integrity

The following procedures were used to assure the integrity of the implementation of the protocol. First, the teachers implementing sessions were provided with a rubric for each session that included the elements and activities for each session to help monitor the steps for each session. Each of the activities planned for the session was detailed in topics, and teachers were asked to check each one off when the activity was completed. Second, on a random basis, two research assistants observed 30% of the sessions using the same rubric used by teachers. These research assistants also wrote a short diary describing teachers’ adherence to the protocol. Third, for the duration of the intervention, on a weekly basis, the principal investigator met with the research assistants to analyze project issues and adherence to protocol of each condition (e.g., analysis of record sheet data). Treatment fidelity was high for the program sessions. Teachers’ reported adherence to the protocol was 93% (*SD* = 2.84, range 90–100). Data from the observations of the intervention sessions indicated that the teachers completed 94% of the activities (*SD* = 3.23, range 87–98).

#### Procedure and Intervention Program

##### Theoretical Framework of the Rainbow Program

The Rainbow Program is rooted in the PLEJE Model of Rosário et al. (2017a,b), which is based on Zimmerman’s (2000) SRL cyclical model. According to Zimmerman (2008), SRL develops over three cyclical phases that describe students’ efforts to prepare the task, perform, and use outcomes to make subsequent adaptations. The forethought phase is anticipatory and comprises learning (e.g., task analysis, such as setting goals and strategic planning) and motivational processes (e.g., self-efficacy and intrinsic interest). These processes occur prior to students’ engagement with the learning tasks and guide their efforts to self-regulate their learning. Prior to the learning task, students set plans regarding specific outcomes they expect to attain and choose the learning strategies likely to help them attain those goals. These processes depend on students’ sources of self-motivation (e.g., self-efficacy). The performance phase or volitional phase describes processes that occur during learning and that affect students’ focus and performance. There are two major categories involved in this phase while approaching a task: use of self-control methods likely to improve performance (e.g., self-instruction, environmental structuring) and self-observation methods (i.e., metacognitive monitoring and self-recording). The latter methods help students to track the use of learning processes and their efficacy and to create formal records of learning outcomes. The self-reflection phase describes students’ reactions to learning outcomes resulting from their efforts to learn. This phase comprises two categories: self-judgments, which refers to students’ comparisons of their performance with a standard (e.g., prior levels of performance) and self-reactions (i.e., self-satisfaction and adaptive/defensive decisions). Self-satisfaction involves students’ cognitive and affective reactions to their self-judgments, while adaptive decisions comprise students’ capacity to make further efforts to continue learning (e.g., maintaining the use of or modifying the strategy used) (Zimmerman, 2008). By contrast, students make defensive decisions to avoid learning experiences and future dissatisfactions (e.g., procrastination, task avoidance) (Rosário et al., 2009). This last phase of the process therefore informs the subsequent forethought phase that completes the self-regulatory cycle. SRL phases are intertwined and the length of the self-regulatory cycle for each student depends on the aspects (personal or environmental, for example) that intervene in the learning process (Bandura, 1986; Zimmerman, 2008).

##### Characteristics and Structure of the Program

The Rainbow Program uses the narrative of “Yellow’s Trials and Tribulations,” designed for children under the age of 10 (Rosário et al., 2007, 2017a,b), and consists of 12 50-min sessions delivered on a weekly basis (see Núñez et al., 2022). This narrative recounts the adventures of the colors of the rainbow while searching for Yellow, who has suddenly disappeared from the rainbow. During this adventure, the colors learn useful SRL strategies to help them overcome obstacles and attain their goals. Through reading and discussion of the story, and with the help of educators, students are encouraged to learn and transfer this knowledge to their daily activities (Rosário et al., 2017a,b).

We present an overview of the role played by three main components of metacognition – knowledge, monitoring, and control – in helping students enrolled in the program to improve their SRL processes. Metacognitive knowledge informs the subsequent elements of metacognition monitoring and control, while also being influenced by these two functions (Winne and Hadwin, 1998). Metacognitive monitoring addresses the progress acknowledged by students while learning. For example, while doing their homework, students are expected to be able to make an inference about the domain of their learning contents and therefore make adjustments to the learning strategies used to complete the homework. Metacognitive control is a form of cognitive control that is informed by metacognitive knowledge or monitoring. With regard to the control of homework tasks, students are expected to balance the time allocated to the importance or difficulty of the task, dedicating more time to complete exercises that are more complex, for example, or changing an SRL strategy when an earlier choice proved to be inefficient (e.g., to focus on the task at-hand and avoid distractors, students may turn off phone notifications or WhatsApp alerts while doing homework). In each session, grounded in discussions about the story plot and the characters’ behaviors, students are encouraged to learn and use the three components of metacognition. For example, metacognitive knowledge is enhanced when students think about their ability to perform a particular task or about the set of SRL strategies they could use to perform that task. Moreover, discussions in class and the activities in the program help students to monitor their efforts to improve learning and the progress achieved but also to control whether their learning efforts are producing the desired learning outcomes.

The sessions proceeded as follows: (a) presentation of the session content; (b) reading from the narrative “Yellow’s Trials and Tribulations,” which, depending on the session, was performed by the teacher for the class, by one child for the class, individually in silence, or collectively with children taking turns to do the reading; (c) completion of a comprehension sheet about the reading with open, closed, direct, and inferential questions; (d) use of SRL strategies to complete short tasks; (e) checking of the homework assigned in the previous session and revision of the content delivered in the previous session; and (f) summary of the current session highlights and setting of homework.

#### Instructional Procedures: Control and Experimental Conditions

The intervention was carried out in the Spanish language class on a weekly basis using one of the four mandatory hours assigned to this subject in the curriculum. Students in the experimental group were provided with the contents of the national curriculum for Spanish (e.g., components of reading, writing, grammar, spelling, and vocabulary). These contents were delivered in 3 h each week, the fourth hour (the last Spanish class of the week) was focused on the activities of the Rainbow Program. Teachers were instructed to apply the SRL strategies discussed in the story tool to the Spanish language content learned during the week. Students enrolled in the control group followed the Spanish curriculum contents for the 4 h each week. Teachers in the control group were instructed to follow the regular Spanish curriculum to meet third- and fourth-grade-level expectations. Thus, the experimental group (Spanish curriculum + SRL training) differed from the control group (Spanish curriculum only) in that, in addition to the usual instruction, the former received training in SRL activities for 1 h a week.

### Data Analysis

Multivariate and univariate likelihood-based mixed-effects regression models (MRMs) was used in the analysis of data. The MRM modeling approach provides an appropriate general analytical framework to determine whether a change in response profiles over time is different between treatment groups and facilitates the comparison of treatment groups in particular time frames. We therefore conducted sensitivity analyses via pattern-mixture models and shared-parameter models in order to explore the impact of deviations from the MAR assumption on the conclusions. In the current analysis, time was treated as a quantitative variable (i.e., measured in months beginning at 0 months for the baseline assessment) rather than as a classification variable. We analyzed the dataset using MRM with maximum likelihood (ML) estimation as implemented in SAS PROC MIXED (SAS Institute Inc, 2018) and the most general mixed model using SAS PROC NLMIXED if the mechanism of missingness was not completely random (MCAR). In addition, we calculated Cohen’s *d* as a measure of standardized effect size using the approach described by Vallejo et al. (2019) for growth curve models with attrition.

Initially, we modeled the effect of the intervention considering four different conditional growth models in competition; each statistical model expanded on a prior model in some logical way. In the first option (Model A), we analyzed data assuming that the 41 classes were assigned to the treatment groups and measured across three time points for four dependent variables. In this first option, the variable class was not included in the random part of the conditional growth model, so the analysis was conducted while ignoring clustering in the data at the classroom level. In the analyses of the second, third, and fourth options (Model B, Model C, and Model D), we analyzed data from 761 students nested in 41 arbitrarily selected classes from 15 middle schools, with the restriction that 20 or 21 classes were randomly assigned to each type of treatment and measured across time in four dependent variables. The three-level conditional Model B examined the effects of different characteristics of the participants at level 2, or the student level (i.e., students’ gender, students’ SRL, and students’ reading comprehension). The three-level conditional Model C added four explanatory variables measured at level 3 or the class level (i.e., current intervention, teacher’s gender, teacher’s experience, and class size). Model D represents a significant simplification over Model C by removing two predictors. The three-level model described provides a way to empirically assess the influence of the class on the observations of the student. If the class effect is observed to be negligible, then analysis using the two-level model for longitudinal data is appropriate; otherwise, the results from the two-level model may be misleading.

After selecting the most parsimonious model, without ignoring any relationships between the outcome variables, we focused on testing the effects of the fitted model. As will be shown later, after controlling for the effects of level-2 and level-3 time-invariant predictors, the multivariate time effect and treatment-by-time interaction were statistically significant. Thus, the next step was to probe the data further to interpret the nature of the specific differences, especially those related to the interaction effects. To this end, we concentrated on least-squared means and pairwise comparisons of differences between the treatment groups at the evaluated time points.

## Results

Observed outcome means, standard deviations, and sample sizes across the four study time points are not provided for reasons of parsimony. These results are available from the first author on request. It is important to note that although the total number of subjects in this study was 761, the number of subjects with all measures at each of the evaluations fluctuated slightly. To test whether the missing data on each of the dependent variables were MCAR, we applied Little’s test (Little, 1988). These data suggest that the MCAR model provides an adequate fit for the data of all dependent variables [χ^2^(4) = 8.34, *p* > 0.05 for observed measurement time points for science; χ^2^(3) = 6.78, *p* > 0.05 for Spanish language; χ^2^(3) = 5.50, *p* > 0.05 for English language; χ^2^(2) = 2.69, *p* > 0.05 for mathematics]. This was further confirmed by examining a plot of estimates as a function of the time of dropout.

### Fitting Competing Models

Table 1 shows the results from the three types of multivariate MRM (i.e., Models A, B, C, and D). Model D was chosen as our “final model” after assessing model fit with likelihood-based AIC and BIC criteria. Empirical results presented by Vallejo et al. (2011) showed the appropriateness of ML for selecting the best mean structure using information criteria. We reached a similar conclusion when comparing the three models using likelihood ratio tests. The deviance statistic and number of estimated parameters in parentheses for Models A, B, C, and D were 10307.7 (38), 10245.3 (39), 10130.3 (63), and 10135.2 (55), respectively. The likelihood ratio test comparing Model B to Model A indicated that Model B was a significantly better fit to the data than Model A was [χ^2^(1) = 62.4, *p* < 0.0001]. When comparing Model C against Model B, the likelihood ratio test indicated that Model C provided a better fit [χ^2^(24) = 115, *p* < 0.0001], while comparing the three-level longitudinal Models D and C, we found a difference in deviance of 4.7 on 8 *df*, which is less than the associated 0.05 critical value of 15.51 (*df* = 8). Model D is a simplification of Model C in which the effects of students’ SRL and teacher experience were removed; we therefore adopted Model D as our final model. These findings provide an argument for using a three-level analysis with within-student measurements at level 1, between-student measurements within classes at level 2, and between-classes measurements at level 3. In addition, because the classes were randomized to study conditions, one could argue that the unit of assignment must remain in the model regardless of significance.

**TABLE 1:** Results of fitting four multivariate mixed-effects regression model analyses.

	Model A		Model B		Model C		Model D	
Fixed effect	*F*-value	Pr > F	*F*-value	Pr > F	*F*-value	Pr > F	*F*-value	Pr > F
LB_Sciences	*F*_4_,_3520_ = 106.0	<0.0001***	*F*_4_,_3510_ = 107.5	<0.0001***	*F*_4_,_3515_ = 107.7	<0.0001***	*F*_4_,_3515_ = 103.4	<0.0001***
LB_Language	*F*_4_,_3524_ = 85.4	<0.0001***	*F*_4_,_3507_ = 99.0	<0.0001***	*F*_4_,_3502_ = 97.9	<0.0001***	*F*_4_,_3502_ = 98.9	<0.0001***
LB_English	*F*_4_,_3522_ = 137.4	<0.0001***	*F*_4_,_3508_ = 134.4	<0.0001***	*F*_4_,_3509_ = 127.9	<0.0001***	*F*_4_,_3502_ = 131.4	<0.0001***
LB_Math	*F*_4_,_3521_ = 87.6	<0.0001***	*F*_4_,_3512_ = 82.9	<0.0001***	*F*_4_,_3515_ = 82.1	<0.0001***	*F*_4_,_3516_ = 82.7	<0.0001***
RC	*F*_4_,_3521_ = 2.9	0.0215*	*F*_4_,_3513_ = 2.0	0.0870	*F*_4_,_3518_ = 2.1	0.0789	*F*_4_,_3517_ = 2.1	0.0824
SRL	*F*_4_,_3521_ = 1.2	0.3222	*F*_4_,_3517_ = 1.2	0.3331	*F*_4_,_3511_ = 1.2	0.3033		
Gender_S	*F*_4_,_3522_ = 9.4	<0.0001***	*F*_4_,_3564_ = 9.2	<0.0001***	*F*_4_,_3574_ = 9.4	<0.0001***	*F*_4_,_3471_ = 9.0	<0.0001***
Gender_T					*F*_4_,_339_ = 7.0	<0.0001***	*F*_4_,_334_ = 7.6	<0.0001***
Experien_T					*F*_4_,_309_ = 0.2	0.9537		
Size Class					*F*_4_,_443_ = 6.4	<0.0001***	*F*_4_,_445_ = 6.6	<0.0001***
Group					*F*_4_,_3218_ = 3.1	0.0146*	*F*_4_,_3266_ = 3.2	0.0117*
Group × RC					*F*_4_,_3505_ = 6.6	<0.0001***	*F*_4_,_3508_ = 6.9	<0.0001***
Time	*F*_4_,_5305_ = 5.4	0.0003***	*F*_4_,_5306_ = 5.4	0.0003***	*F*_4_,_5305_ = 5.1	0.0005***	*F*_4_,_5305_ = 5.1	0.0005***
Group × Time					*F*_4_,_5305_ = 5.0	0.0013**	*F*_4_,_5305_ = 5.0	0.0012**

**Random effect**	**Estimate**	**SE**	**Estimate**	**SE**	**Estimate**	**SE**	**Estimate**	**SE**

**Level-1 (within-subject variance)**								
Residual	0.2728***	0.0053	0.2728***	0.0053	0.2665***	0.0052	0.2666***	0.0052
**Level-2 (between students within classes variances)**								
Intercept	0.0863***	0.0063	0.0***	0.0055	0.0684***	0.0054	0.0685***	0.0055
**Level-3 (between-classes variances)**								
Intercept			0.0188***	0.0053	0.0143***	0.0045	0.0144***	0.0045
**Goodness-of-fit**								
Deviance	10307.7		10245.3		10131.3		10135.1	
No. parameter	38.0		39.0		63.0		55.0	
AIC	10387.7		10323.3		10256.3		10245.1	
BIC	10559.7		10391.1		10365.8		10340.8	

*LB_Sciences, baseline sciences performance; LB_Language, baseline Spanish language performance; LB_English, baseline English language performance; LB_ Math, baseline mathematics performance; SE, standard error. RC, students’ reading comprehension; students’ self-regulation. Gender_S, students’ gender; Gender_T, teachers’ gender; Experien_T, teachers’ experience.*

*p < 0.05; **p < 0.01; ***p < 0.001.

Comparing the variance component estimates for level 2 and level 3 in Model D to those of the three-level unconditional growth model, we found a decline of 0.8516 and 0.0286, respectively. In other words, 92.6% of the between-student variation in outcomes measured at different time points was explained by the covariates at the student level, whereas 65.2% of the between-class variation in outcomes measured at different time points was explained by the covariates at the class level and cross-level interaction terms (i.e., treatment by linear trend and treatment by reading comprehension). Moreover, an additional 18.5% of within-subject variation in outcomes was explained by linear time.

### Multivariate Mixed-Effects Regression Model Analyses

Inspection of Table 1 reveals the following. First, students’ gender showed a statistically significant effect on all dependent variables considered simultaneously [*F*(4,3471) = 9.03, *p* < 0.001]. Findings indicated that girls outperformed boys in overall academic achievement. In addition, the interaction term of students’ reading comprehension × treatment groups showed significant effects on dependent variables [*F*(4,3508) = 6.92, *p* < 0.001], indicating that the intervention program was more beneficial to children with low reading comprehension skills than to children with high reading comprehension skills. Second, teacher’s gender and class size had a statistically significant effect on all dependent variables considered simultaneously [*F*(4,333) = 7.57, *p* < 0.0001; *F*(4,445) = 6.65, *p* < 0.001]. Specifically, the academic achievement of students in the control group classes with male teachers was higher than that of students in classes with female teachers. In the experimental group, the results indicated the opposite trend; children in classes with female teachers showed slightly higher scores than children in classes with male teachers. The results also indicated that students in classes with fewer students performed better than their counterparts in larger classes. Third, averaged across treatment groups, there was a significant [*F*(4,5305) = 5.05, *p* < 0.001] increase in the mean response over time by simultaneously considering all dependent variables; to put it another way, on average, participants improved over time. Fourth, it is very important to note that, controlling for the effects of covariates, there was a significant difference between the treatment conditions over time in the set of the four dependent variables considered simultaneously [*F*(4,5305) = 4.47, *p* < 0.01]. Acknowledging that the interaction was significant (i.e., the pattern of change in the variables measured over time was not similar in both groups), we focused our attention on this finding. The differences of least square means in Table 2 provide pairwise comparisons between the treatment groups over time. More specifically, the results showed that there were significant differences between the treatment and control group means, both at the end of treatment (i.e., second evaluation) and 3-month follow-up (i.e., third evaluation).

**TABLE 2:** Comparisons of group × time least-squares means by simultaneously considering all dependent variables.

Effect		Time	Estimate	SE	*df*	*t-*value	Pr > | *t*|	*d*
Group × Time	CG vs. EG	Post-test	−0.5541	0.161	1714	−3.44	0.0006***	0.25
Group × Time	CG vs. EG	Follow-up	−0.6974	0.161	1714	−4.42	<0.0001***	0.33

*Group, control vs. experimental; Time, measurement time points; SE, standard error; df, degree of freedom.*

According to Cohen’s guidelines, d values of 0.2, 0.5, and 0.8 are considered small, medium, and large effect sizes, respectively. *p < 0.05; **p < 0.01; ***p < 0.001.

### Univariate Mixed-Effects Regression Model Analyses for Each Dependent Variable

We conducted follow-up univariate MRM analyses to determine which dependent variables were responsible for the significant omnibus test of group-by-time interaction. Table 3 includes results of the hypothesis tests for the outcome response measurement data.

**TABLE 3:** Results of mixed-effects regression analysis of each of the four dependent variables.

Fixed effects					Random effects				
Effect	df_*N*_	df_*D*_	*F*-value	Pr > F	VC	Estimate	SE	*Z*-value	Pr > Z
***Natural Sciences***									
LB	1	756	1602.39	<0.0001***	σ^2^	0.1967	0.0101	19.44	<0.0001
Gender_S	1	747	0.12	0.7311	τ_001_	0.1839	0.0158	11.66	<0.0001
RC	1	754	22.41	<0.0001	τ_002_	0.0223	0.0085	2.64	0.0041**
Gender_T	1	43	2.15	0.1503					
Class size	1	63	4.28	0.0427*					
Group	1	764	3.49	0.0620					
Group × RC	1	756	8.21	0.0043**					
Time	1	756	2.37	0.1240					
Group × Time	1	756	9.87	0.0017**					
***Spanish Language***									
LB	1	733	1898.58	<0.0001	σ^2^	0.1474	0.0076	19.34	<0.0001
Gender_S	1	737	5.23	0.0225*	τ_001_	0.1187	0.0110	10.82	<0.0001
RC	1	744	24.95	<0.0001	τ_002_	0.0168	0.0062	2.71	0.0034**
Gender_T	1	42	2.00	0.1643					
Class size	1	60	0.44	0.5108					
Group	1	762	0.23	0.6281					
Group × RC	1	748	0.02	0.8781					
Time	1	748	2.88	0.0899					
Group × Time	1	748	10.65	0.0012**					
***English***									
LB	1	759	1368.50	<0.0001	σ^2^	0.2134	0.0110	19.47	<0.0001
Gender_S	1	747	0.12	0.7334	τ_001_	0.2567	0.0200	12.85	<0.0001
RC	1	757	16.41	<0.0001	τ_002_	0.0311	0.0117	2.66	0.0039**
Gender_T	1	41	0.04	0.8335					
Class size	1	60	3.23	0.0774					
Group	1	729	2.28	0.1315					
Group × RC	1	759	4.50	0.0341*					
Time	1	759	5.35	0.0210*					
Group × Time	1	759	5.93	0.0149*					
***Mathematics***									
LB	1	749	1416.73	<0.0001***	σ^2^	0.1984	0.0102	19.42	<0.0001
Gender_S	1	736	1.27	0.2596	τ_001_	0.1819	0.0158	11.54	<0.0001
RC	1	753	22.12	<0.0001	τ_002_	0.0459	0.0137	3.35	0.0004***
Gender_T	1	45	0.27	0.6080					
Class size	1	58	0.06	0.8122					
Group	1	662	0.06	0.8080					
Group × RC	1	749	0.29	0.5876					
Time	1	755	16.97	<0.0001					
Group × Time	1	755	0.04	0.8448					

*LB, baseline academic performance; RC, students’ reading comprehension; Gender_S, students’ gender; Gender_T, teachers’ gender; Group, control vs. experimental; Time, measurement moments; VC, variance component (σ^2^, within-subject variance; τ_001_, between students within classes variance; τ_002_, between-classes variance).*

*p < 0.05; **p < 0.01; ***p < 0.001.

The data in Table 4 indicate that, except for student achievement in mathematics [*F*(1,754) = 0.04, *p* > 0.05], the null hypothesis of no differences between treatment conditions with respect to their average growth rates is rejected at a level of significance of no more than 1.5% for all outcome variables [*F*(1,756) = 9.87, *p* < 0.01; *F*(1,748) = 10.65, *p* < 0.01; *F*(1,759) = 5.93, *p* < 0.05]. In their entirety, these data indicate the efficacy of the intervention when considering the observation time point. Current data show that the time of implementation of the program is crucial to judging the efficacy of the intervention.

**TABLE 4:** Comparisons of group × time least-squares means for each dependent variable (subject) and theirs standardized effect size.

		Natural Science		Spanish		English		Mathematics	
Group	Time	Estimate (SE)	*d*	Estimate (SE)	*d*	Estimate (SE)	*d*	Estimate (SE)	*d*
CG vs. EG	Post-test	−0.052 (0.067)	0.08	−0.058 (0.057)	0.11	−0.078 (0.075)	0.11	0.091 (0.089)	0.12
CG vs. EG	Follow-up	−0.195** (0.067)	0.31	−0.187** (−057)	0.35	−0.194* (0.075)	0.28	0.085 (0.089)	0.11

*CG, control group; EG, experimental group.*

*According to Cohen’s guidelines, d values of 0.2, 0.5, and 0.8 are considered small, medium, and large effect sizes, respectively.*

*p < 0.05; **p < 0.01.

**FIGURE 1:**
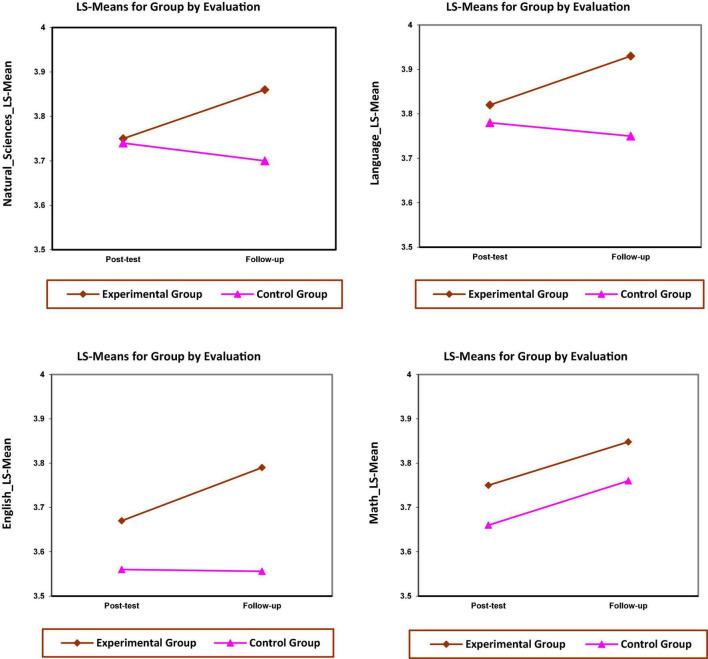
Interactions plots: least-squares means over time by groups for each type of dependent variable (i.e., Sciences, Spanish language, English language, and Mathematics).

The next step aims to explain the group-by-time interaction in the response variables in a manner consistent with our research objectives. We estimated and compared linear combinations of means for this purpose using the LSMEANS statement in PROC MIXED. The least-squares means are estimates of the two groups evaluated at the end of treatment (i.e., second evaluation) and 3-month follow-up (i.e., third evaluation) for each dependent variable. These means are graphed in Figure 1.

As one would expect, there was a delay before the experimental treatment started to exhibit a beneficial effect in the school subjects (science, Spanish language, and English language). Table 4 summarizes the results of the analysis. In this same table, following the approach by Vallejo et al. (2019), we report Cohen’s *d* local effect sizes for group-by-time interaction effects as appropriate for multilevel modeling analysis. These values were calculated separately for the end of treatment (i.e., second evaluation) and the 3-month follow-up (i.e., third evaluation).

## Discussion

Knowledge about and training in strategies for SRL are likely to help improve the quality of learning and performance in various academic areas (De Bruijn-Smolders et al., 2016). Data from meta-analyses (e.g., De Boer et al., 2018) agree that the most effective intervention programs have a metacognitive and social-constructivist background and are delivered by researchers. However, the results of previous research are not consistent when it comes to long-term effects or which class-level variables (e.g., teacher’s gender, teacher’s experience, and class size) influence the efficacy of the interventions.

In general, data from our study show three results. First, a positive effect of the intervention on student performance, both at post-test (*d* = 0.25) and at follow-up (*d* = 0.33), when the four school subjects are considered together. However, the effect is only significant at follow-up (3 months after the end of the intervention) if the subjects are considered separately. Second, student performance is significantly related to the students’ variables (i.e., gender, level of reading comprehension) and the context (teacher’s gender and class size). Third, the student’s gender, the student’s level of reading comprehension, and the teacher’s gender are moderators of the effect of the intervention on students’ academic performance. We discuss these results in more detail below.

### Post-test Effects

In our first hypothesis, considering the data reviewed, we proposed that, for the 3-month post-intervention, (1) the academic performance of the students in the experimental group would be statistically higher than that of the students in the control group; (2) those differences would be similar in the four subject areas examined; and (3) the size of the effect would be moderate (approximately between 0.40 and 0.50). Overall, data from the present study do not fully support this hypothesis.

As noted above, without differentiating between the different academic areas, the intervention showed a statistically significant effect on student performance (mean performance) at post-test, although with a small effect size (*d* = 0.251). However, if we consider each of the subjects individually, although students enrolled in the experimental group increased their performance scores in the four areas (while the control group maintained their scores), the differences between the two groups did not reach statistical significance. Consequently, unlike the predictions based on the reviewed meta-analyses (e.g., Dignath et al., 2008) and despite the positive trend found [science (CG = +0.06, EG = +0.10); Spanish language (CG = +0.02, EG = +0.15); English language (CG = +0.03, EG = +0.12); mathematics (CG = −0.03, EG = +0.04)], the effect size for each of the subjects was not statistically significant. How might we explain this finding?

Several variables may help explain these findings. For example, the students’ educational needs (regular vs. special needs) may be an important variable to explain the disparity of the effect sizes found in interventions and, in part, the results of our study. Generally, interventions with students with special needs, when compared with those of students without special needs, are more effective. For example, very large effect sizes have been reported in students with learning difficulties in writing (e.g., Mourad, 2009, *d* = 2.55) and with difficulties in mathematics (e.g., Pennequin et al., 2010, *d* = 2.17). Literature reported large effect sizes in studies on difficulties in reading (e.g., Mason, 2004, *d* = 0.92) and difficulties in writing (e.g., Graham et al., 2005, *d* = 0.92). In addition, to the best of our knowledge, only a few interventions with students with special needs have reported moderate effect sizes (e.g., Wright and Jacobs, 2003, *d* = 0.68). By contrast, there are many more studies with students without disabilities reporting null and minimal effect sizes (e.g., Allen and Hancock, 2008, *d* = 0.15; Meyer et al., 2010, *d* = 0.08; Souvingnier and Mokhlesgerami, 2006, *d* = 0.14; Van Keer and Vanderlinde, 2010, *d* = 0.05) or small effect sizes (Stoeger and Ziegler, 2008, *d* = 0.36; Tracy et al., 2009, *d* = 0.34) than those that have reported larger effect sizes (e.g., Michalsky et al., 2009, *d* = 0.79). The effect sizes of the current intervention at post-test are very small (science: *d* = 0.08; Spanish language: *d* = 0.11; English language: *d* = 0.11; mathematics: *d* = 0.00), but they are similar to those of the aforementioned research. It is possible that working on macro strategies (e.g., planning, monitoring, and evaluation) with students without specific needs is a strategy with a longer term payoff, particularly if the instructional environment allows them to practice and improve. This is what seems to have happened in our study when we analyze the post-test data.

Another potential explanation for the small effect of the SRL intervention on academic performance (in the post-test) could be the limited mediational effect of SRL, as suggested by the meta-analysis data of Jansen et al. (2019) or Núñez et al. (2022). SRL interventions are designed to improve knowledge of SRL strategies and perceived competence (Núñez et al., 2013; Cerezo et al., 2019), metacognition (Dignath et al., 2008), and student involvement in SRL activities (De Bruijn-Smolders et al., 2016), which is likely to lead to an improvement in performance (Dent and Koenka, 2016). However, data from the recent meta-analysis by Jansen et al. (2019) indicate that SRL partially mediates the relationship between SRL interventions and academic performance. Specifically, findings indicate that the “indirect effect of SRL interventions on achievement is small, and that a significant direct effect of SRL interventions on achievement remains after including SRL activity as a mediator” (Jansen et al., 2019, p. 14). Thus, recent findings indicate that SRL activity is a partial mediator of the effect of SRL interventions on achievement. Although this may seem puzzling, the effect of SRL interventions on performance are mediated by several variables beyond the SRL activity, both at the individual and class level (e.g., students’ self-efficacy, students’ academic procrastination, opportunities to use SRL in class to solve exercises, and type of assessment delivered).

### Follow-Up Effects

Our second study hypothesis stated that (i) the effect of the intervention would be maintained or even increase 3 months after the post-test (follow-up) and (ii) the effect would be similar in the four academic areas. Our data partially confirm the hypothesis, although the effect size was smaller than expected. When we consider students’ performance as a whole, without differentiating the subject areas, the effect of the intervention was significant and positive (*d* = 0.325), and even increased in the post-test [dif(follow-up-post-test) = 0.074]. However, when analyzing the effect of the intervention for each of the subjects separately, we can see statistically significant differences, with the experimental group scoring higher than the control in three of the four areas (science, Spanish language, and English language) although the effect size was small (science: *d* = 0.31; Spanish language: *d* = 0.35; English language: *d* = 0.27). We did not see significant effects from the intervention in mathematics. In general, our data are in line with findings from the meta-analysis by De Boer et al. (2018), as long as we limit the analysis to studies with students of a similar age (e.g., Tracy et al., 2009; Brunstein and Glaser, 2011; Carretti et al., 2014; Stoeger et al., 2014). De Boer et al. (2018) reported statistically non-significant differences between areas or domains with respect to the difference between the intervention effects at post-test and follow-up.

How can we explain current results for mathematics? Why did the mathematics results fail to improve 3 months post-intervention, following a trend similar to that of the other subjects? One possible explanation may be the distinct ways in which teachers understand the subject and organize activities in class. These distinct approaches to the subject are likely to influence students’ SRL. For example, Wolters and Pintrich (1998) found that teachers’ beliefs about the nature of their subject influenced instructional practices. Extant literature has shown that mathematics is often perceived by teachers as a very defined, sequential, and not very dynamic subject, whereas languages (e.g., English, Spanish) or social studies are considered to be much more open and dynamic (Dent and Koenka, 2016). This finding may help explain the highly structured choice of mathematical tasks (with very clear procedural content, concrete answers, and precise evaluation criteria) to be delivered in class (Lodewyk et al., 2009). This sequence holds within the characteristics of an SRL approach (e.g., design a plan, establish sub-goals, use monitoring strategies, project a foreseeable result), which may prevent the development of students’ autonomy and personal agency. In sum, instructional processes developed in mathematics classes, understood as sequential and static, can limit the use of SRL activities in class and, therefore, weaken their association with achievement (Lodewyk et al., 2009). By contrast, the use of less structured tasks in class, typical of subjects such as Spanish language or English language, require the use of metacognitive processes to further define and structure the activities and achieve success in completing them. In this way, the use of this type of task in class is likely to encourage students to use metacognitive and SRL strategies (Lodewyk et al., 2009). This line of reasoning is consistent with data from the meta-analysis by Dent and Koenka (2016), who found a stronger and significant association between metacognitive processes and performance in social studies than in mathematics.

The second aspect of interest is related to the effect size of the SRL intervention. Unfortunately, we did not gather data on the class dynamics; these data would have helped understand whether the instructional scenarios developed in the 3 months post-intervention promoted or prevented the use of metacognitive resources and SRL strategies in class. As suggested by Paris and Paris (2001), for SRL interventions to achieve the expected educational impact, it would be necessary to intervene in the variables of the instructional context (e.g., type of feedback and type of assessment delivered). Therefore, regardless of subject, for SRL interventions to be truly successful (to have a large effect on learning and performance), the metacognitive and SRL strategies conveyed would likely need to be intertwined with daily classroom activities. In this way, teachers and students would have the opportunity to practice, apply, and extend their metacognitive knowledge but also their metacognitive monitoring and control, improving students’ performance as a result. As Paris and Paris (2001, p. 96) have noted, “SRL may be regarded not as the goal of students’ learning but as the outcome of their pursuits to adapt to their unique environmental demands in a coherent manner.” In sum, teachers are expected to promote opportunities in class for students to use metacognition and SRL processes and help them grow and develop positively at school.

### Potential Moderators of the Effectiveness of Self-Regulated Learning Strategy Interventions

#### Student Characteristics

In our study, we included student gender alongside levels of initial reading comprehension and SRL strategies (pre-test) as covariates. The data from the analyses indicate that student gender and initial reading comprehension level were significantly related to student performance: the size of the effect of gender was small (*d* = 0.23), and the size of the effect of reading comprehension was marginal (*d* = 0.11). More specifically, girls showed significantly better school results than boys, as did students with higher levels of reading comprehension compared to those with lower levels. However, the use of SRL strategies did not show a significant effect on performance.

Nevertheless, we had a dual interest: studying the interaction of these variables with the intervention and examining how they might moderate the effect of the intervention on performance (both overall and individually). The data show a significant moderating effect of reading comprehension (*d* = 0.20) and a marginal moderating effect of student gender (*d* = 0.11). We found no notable interaction between the levels of SRL strategies and the intervention.

The interaction of reading comprehension and the intervention showed that students with lower levels of (pre-test) reading comprehension benefited more from the intervention than did students with higher levels. This is consistent with the findings of some studies (e.g., Stoeger and Ziegler, 2010) but not with those of others (e.g., Morgan et al., 2011; Sontag and Stoeger, 2015; Otto and Kistner, 2017). Otto and Kistner (2017) suggested that their results, consistent with the Matthew effect and in contrast to what might be expected (that greater gains would be made by those starting from lower levels) (De Corte et al., 2011), may have been due, at least in part, to the short duration of the intervention (five sessions of effective work). They also suggested that longer interventions might allow students with a lower level to have sufficient time to maximize their gains even more than their counterparts with higher initial levels. The results of our 12-session study are consistent with the hypothesis put forward by Otto and Kistner (2017), but researchers may wish to examine the hypothesis further.

Otto and Kistner also suggested that their results might have been due to the content of their training program. In the current study, the students were trained in macro strategies within a socio-cognitive framework (planning, monitoring, and evaluation) applied to general tasks in the learning process and to the specific context of text comprehension. It is possible that students with lower reading comprehension were in more need of this type of training, which did not require high cognitive abilities for them to benefit from it.

Student gender, without differentiating between the four subjects, was shown to be statistically significantly related to student performance (small effect; *d* = 0.23), and to exhibit a marginal interaction with the intervention (irrelevant effect; *d* = 0.11). Running the model for each subject shows that student gender had a small effect with performance in Spanish language (*p* = 0.022). Moreover, gender was not found to be a moderating variable for any of the four subjects.

Overall, the current data do not show an effect of student gender on performance. Moreover, changes in academic performance associated with the intervention are basically parallel (the effect of the intervention was similar for performance in the post-test and follow-up).

#### Context Characteristics

Teacher’s gender, teacher’s experience, and class size were included in the model as variables at the class level. The data indicate that teacher’s gender and the class size influenced student performance (*d* = 0.14 and *d* = 0.15, respectively) but that teaching experience did not. More specifically, we learned that although the effect size was small in both cases, students had better (average) results when they learned with male teachers and when they were enrolled in small classes. However, when the school subjects were considered separately, we did not find a significant effect of teacher’s gender on performance in any of the subjects, but class size showed a small effect in science and a marginal effect in English language. These results, in short, seem to suggest that none of the three variables (i.e., teacher’s gender, teaching experience, and class size) significantly explains the differences in student performance, particularly when subjects are considered separately.

Analyzing the effect of the interaction of these three variables with the intervention, we found that results were significantly better when students were taught by female teachers. In addition, neither the amount of teaching experience nor the size of the class was shown to be related to the effects of the intervention. In other words, the effect of the intervention on student performance was independent of the teacher’s experience and the class size, although it was enhanced when the teacher was female.

With regard to teacher characteristics, current data is consistent with data from international studies with fourth graders investigating similar subjects (e.g., Luschei and Chudgar, 2011; Blömeke and Olsen, 2019). For example, Luschei and Chudgar (2011) examined data from the 25 countries that participated in TIMSS – 2003. They analyzed the relationships between teacher characteristics (experience, education, readiness to teach, and gender), student background, and fourth-grade students’ mathematics and science performance. Their results indicated that the impact of teacher characteristics on student performance is limited. More recently, Blömeke and Olsen (2019) used the TIMSS – 2001 database to examine the academic achievement in mathematics and science of fourth-grade students from the United Kingdom, Norway, South Korea, Thailand, and Tunisia. The findings from this study were consistent with those of Luschei and Chudgar (2011), indicating that the relationship between teacher characteristics and student achievement is weak.

In summary, the current results are in line with data from the large-scale studies mentioned above. Contrary to our initial expectations, teacher experience does not play a relevant role in the explanation of the variability in students’ performance.

## Conclusion

Our results indicate that the intervention is effective in relation to the two measuring time points (post-test and follow-up) and the four academic subjects taken together. When subjects were considered separately, statistically significant differences between experimental and control groups were found in three of the four subjects examined (mathematics being the exception). However, the absence of effect in mathematics may be due to the characteristics of the tasks and the instructional process of the discipline itself (Dent and Koenka, 2016), but also possibly due to the improvement experienced by the control group (beyond what was expected). Future research may wish to examine these possibilities.

The results of our study emphasize the importance of considering time in intervention studies. Researchers may wish to consider including this variable in future work. For example, they may wish to examine the relationship between intervention characteristics and the time needed to observe significant or notable changes (Núñez et al., 2013). In this regard, intervention studies could consider the inclusion of additional measures besides pre-test and post-test (e.g., Rosário et al., 2019). Moreover, the current results indicate that teaching experience is not necessarily related to progressive improvements in the quality of students’ instruction. Indeed, Blömeke and Olsen (2019) examined teaching experience and teaching quality as individual variables and found that only quality of teaching significantly predicts student performance. Therefore, those responsible for educational policy might wish to consider developing initial and continuing training strategies for teachers (see Egert et al., 2020), allowing teachers to improve their teaching quality while taking advantage of their experience as teachers (Michalsky and Schechter, 2013; Rosário et al., 2013; De Smul et al., 2018; Iwai, 2019; Egert et al., 2020; Frazier et al., 2021; Högemann et al., 2021; Karabenick et al., 2021; Kitsantas et al., 2021). The study by Iwai (2019) provides a good example of the implementation of such a proposal. The author analyzed the effects of training in metacognitive and self-regulation strategies for preservice teachers. Specifically, these future elementary-school teachers were trained to plan, implement, and analyze metacognitive strategies for reading and writing activities. The results showed that after one semester of training, the preservice teachers were able to select and optimally use appropriate metacognitive strategies based on the needs of their students and on the objectives for the lesson (these preservice teachers increased their awareness, knowledge, and skills to use metacognitive strategies). Likewise, they were able to critically analyze their own use of metacognitive strategies.

The data from our study and any educational implications derived therefrom should be interpreted with caution due to important limitations both in the design used and in the SRL evaluation procedure. With regard to the former, despite the large number of students and classes for each of the conditions, the interpretation of the results would benefit from the inclusion of a third group (placebo). To further ground our inferences about the increase in performance by students who received regular instruction within an SRL framework compared to those who received only regular instruction, it would have been valuable to have had evidence of a progressive increase in SRL activities and use of SRL skills accompanying the improvement in performance. For example, Rosário et al. (2017a,b) carried out a study (a longitudinal classroom-randomized controlled design using a multilevel modeling analysis) to examine the impact of extra writing opportunities (i.e., writing journals) on the quality of the writing compositions of 182 fourth-grade students. During the 12 weeks of the intervention, students in the control and experimental conditions wrote a weekly journal. The data indicated that the differences in the quality of the written compositions at the end of the 12th week were modulated by the use of SRL strategies over time. Moreover, the relationship between time (i.e., 12 weeks) and students’ writing performance was found to be quadratic, rather than non-linear; the writing quality of the compositions increased more rapidly and intensively in the first 3 weeks, with the curve presenting a progressive but only slight growth in the subsequent weeks. Finally, while analyzing the latter limitation, we acknowledge the use of self-reporting to measure SRL. Self-reports are not exempt from limitations (regarding reliability and validity) and may not be adequate for measuring a construct of a processual nature, such as SRL (Karabenick and Zusho, 2015; Panadero et al., 2017). Future research may wish to consider using more than one source of information to collect data (Rovers et al., 2019; Järvela et al., 2021).

## Data Availability Statement

The raw data supporting the conclusions of this article will be made available by the authors, without undue reservation.

## Ethics Statement

The studies involving human participants were reviewed and approved by the Comité de Ética de la Investigación en Ciencias Sociales y Humanas de la Universidad de Oviedo. Written informed consent to participate in this study was provided by the participants’ legal guardian/next of kin.

## Author Contributions

ET, JN, and FA conceived the original idea and supervised and coordinated the project and data collection. TM, JM, and PR developed the theoretical framework and design of the study. GV and MF performed the data analysis and findings. All authors discussed the results and contributed to the final manuscript.

## Conflict of Interest

The authors declare that the research was conducted in the absence of any commercial or financial relationships that could be construed as a potential conflict of interest.

## Publisher’s Note

All claims expressed in this article are solely those of the authors and do not necessarily represent those of their affiliated organizations, or those of the publisher, the editors and the reviewers. Any product that may be evaluated in this article, or claim that may be made by its manufacturer, is not guaranteed or endorsed by the publisher.
